# Mapping the *N-*linked glycosites of rice (*Oryza sativa *L.) germinating embryos

**DOI:** 10.1371/journal.pone.0173853

**Published:** 2017-03-22

**Authors:** Jiezheng Ying, Juan Zhao, Yuxuan Hou, Yifeng Wang, Jiehua Qiu, Zhiyong Li, Xiaohong Tong, Zhaomei Shi, Jun Zhu, Jian Zhang

**Affiliations:** 1 State Key Lab of Rice Biology, China National Rice Research Institute, Hangzhou, P.R. China; 2 Jingjie PTM-Biolabs, Hangzhou, P.R. China; The University of Melbourne, AUSTRALIA

## Abstract

Germination is a key event in the angiosperm life cycle. *N*-glycosylation of proteins is one of the most common post-translational modifications, and has been recognized to be an important regulator of the proteome of the germinating embryo. Here, we report the first *N-*linked glycosites mapping of rice embryos during germination by using a hydrophilic interaction chromatography (HILIC) glycopeptides enrichment strategy associated with high accuracy mass spectrometry identification. A total of 242 glycosites from 191 unique proteins was discovered. Inspection of the motifs and sequence structures involved suggested that all the glycosites were concentrated within [NxS/T] motif, while 82.3% of them were in a coil structure. *N*-glycosylation preferentially occurred on proteins with glycoside hydrolase activities, which were significantly enriched in the starch and sucrose metabolism pathway, suggesting that *N-*glycosylation is involved in embryo germination by regulating carbohydrate metabolism. Notably, protein-protein interaction analysis revealed a network with several Brassinosteroids signaling proteins, including XIAO and other BR-responsive proteins, implying that glycosylation-mediated Brassinosteroids signaling may be a key mechanism regulating rice embryo germination. In summary, this study expanded our knowledge of protein glycosylation in rice, and provided novel insight into the PTM regulation in rice seed germination.

## Introduction

Protein glycosylation, which is an important form of co-translational and post-translational modifications (PTMs), refers to the enzymatic, covalent addition of glycans to proteins [1]. Glycosylation of a protein imposes comprehensive effects on its folding, localization, trafficking, solubility, antigenicity and many other aspects, thus endows the modified protein with new functions [2]. Depending on the linkage between the amino acid and glycans, protein glycosylation are divided into four categories, namely *N-*linked glycosylation (*N*-glycosylation), O-linked glycosylation, C-mannosylation and glycosylphosphatidylinositol (GPI) anchor attachments [2,3]. An analysis of the 75,000 glycosylated protein entries in SWISS-PROT estimated that *N-*glycosylated proteins accounted for over two thirds with a frequency of 3.1 sites per protein, representing the richest glycosylation type in eukaryotes [4]. In contrast to the features of O-linked glycosylation of which glycans are usually attached to the hydroxyl group of serine, threonine and tyrosine sites, *N*-glycosylation mostly occurs on the amide of asparagines which are located in a consensus motif NxS/T (x can be any amino acids except proline) [2]. During *N-*glycosylation, oligosaccharide transferases catalyze the link between the lipid-linked oligosaccharide (LLO) precursor and the nascent polypeptide in the endoplasmic reticulum (ER). The proteins harboring high-mannose type *N-*glycan moiety mature at Golgi where *N-*glycan proteins are formed. When the *N-*glycan protein exit golgi, the GlcNAc is cleaved and form paucimannose type *N-*glycan. [5]. Although the cellular machinery used to *N-*glycosylate is well conserved, the capacity to form hybrid or complex *N-*glycans does vary between species and between cell types [6]. *N*-glycosylation is involved in protein folding, ER-associated degradation and protein sorting, processes which underpin growth, development, morphogenesis and stress signaling [3,7–9]. In human, the aberrant status and structure of *N-*glycan in cell surface receptors have been associated with the cancer cell progression and metastasis [10]. Similarly, mutants of genes encoding certain plant *N*-glycosylation enzymes suffer from severe growth defects [8]. In *Arabidopsis thaliana*, for example, some lesions to a gene encoding α-glucosidase I (which catalyzes the first trimming reaction of *N-*glycans) are lethal, while others display dwarfing, foreshortened roots and altered epidermal cell patterning [11,12]. In rice, null mutation of *GnTI* (*N-acetylglucosaminyltransferase I*) caused arrested seedling development, lethality prior to reporduction, defective cell wall synthesis and cytokinin insensitivity [13]. *cgl1* (*complex glycan 1*), which is a mutant of *GnTI* ortholog in Arabidopsis, also exhibited growth inhibition, aberrant root-tip morphology, and callose accumulation under salt stress [14]. In addition to the cases mentioned above, protein *N*-glycosylation has also been implicated in plant immunity to disease, abiotic stress response, low temperature sensing and so on, indicating the essential roles of *N*-glycosylation in plants [14–17].

So far, our current knowledge of *N*-glycosylation is majorly acquired by studying the function of genes which encode key enzymes, while the target glycoproteins which execute biological functions and the networks of glycoproteins have received less attention. To address this question, profiling the glycoproteins and glycosites is certainly fundamental and crucial. A number of platforms are now on hand to study glycoproteins: these include enrichment techniques such as hydrazide capturing, lectin-specific capturing, affinity separation using porous graphite carbon, HILIC (hydrophilic interaction chromatography) or boronic acid nanoparticles [18]. By coupling these technologies with high-accuracy Mass Spectrometry (MS) identification, scientists have successfully profiled a number of plant *N-*glycoproteomes in large scales [19–29].

Rice (*Oryza sativa* L.) is a major food crop as well as a model plant for biological research [30,31]. As is the case of most angiosperms, the life cycle of rice begins with the germination of the embryo. The imbibition of water into a dry grain rehydrates the embryo, which triggers the resumption of its metabolism. Following this, the embryo begins to synthesize a range of proteins required to support its starch metabolism, glycolysis, major carbohydrate metabolism and amino acid metabolism [32–34]. Like other complex physiological processes, rice seed germination is subjected to the regulation of many proteins and PTMs including *N*-glycosylation [35,36]. As yet the extent to which *N-*glycoproteins are of importance in the germination of the rice grain is unknown. To the best of our knowledge, the current study presents the first effort of large scale profiling of *N-*linked glycosites in rice, with a special focus on the germinating embryos. Our result revealed the extensive involvement of *N*-glycosylation in energy metabolism and Brassinosteroids (BRs) signaling during embryo germination, which shed novel light into the mechanisms of PTM regulation in plants.

## Material and methods

### Plant materials and sample collections

Nipponbare (*Oryza sativa* L. *ssp japonica*) seeds were harvested from the field in China National Rice Research Institute. Dehusked seeds of Nipponbare were immersed in distilled water at 28°C for 24 hours. Germinating embryos were manually dissected from the seeds by blades with three biological replicates and immediately kept in liquid nitrogen for future use. For BRs induction, 14-day-old NIP seedlings were spread with 10 μM BRs (Sigma, St Louise, U.S.A.) on leaves and collected at 3, 6, 12 and 24 hours after treatment for RNA isolation (untreated seedlings were taken as control). The embryo samples in the process of germination were collected as mentioned above at the time points including 3 hours, 6 hours, 12 hours and 24 hours for RNA isolation.

### RNA isolation and qRT-PCR analysis

Total RNA of samples were extracted using Trizol (Invitrogen, Carlsbad, U.S.A.) and their corresponding cDNA were reverse transcribed using 2 μg of each RNA by M-MLV reverse transcriptase (Takara, Dalian, China) according to the manufacturer's instructions. To investigate the expression patterns of related genes in BRs induction and embryo germination, real-time fluorescence quantitative PCR was performed by CFX96 touch real-time PCR detection system (Bio-rad, Hercules, U.S.A.). The reaction system was composed of 1 μL cDNA template, 5 μL THUNDERBIRD SYBR^®^ qPCR Mix (TOYOBO, Shanghai, China), 0.2 μL primers (10 μM), and 3.8 μL sterile ddH_2_O with 3 replicates. The expression level of genes was acquired by calculating the 2-^ΔΔ^CT values with comparison to the expression of ubiquitin gene. The sequences of RT-PCR primers used in this study were listed in S1 Table.

### Protein extraction and trypsin digestion

For protein extraction, 2 grams of rice embryo samples were first ground into fine powders in liquid nitrogen, lysised in 15 mL lysed buffer (8 M urea, 10 mM dithiothreitol, 2 mM EDTA and 1% Protease Inhibitor Cocktail) by shaking on ice for 20 minutes, and precipitated with ice-cold 15% TCA. After been washed with cold acetone for three times, the extracted protein was dissolved in 2 mL buffer (8 M urea, 100 mM NH_4_CO_3_, pH 8.0) and quantified by Qubit^®^ 3.0 Fluorometer (Invitrogen, Carlsbad, U.S.A.).

To do the trypsin digestion, protein was sequentially treated with 10 mM dithiothreitol at 37°C for 1 hour and 20 mM iodoacetamide (IAA) for 45 minutes at room temperature in darkness. The alkylated protein was then diluted with 100 mM NH_4_CO_3_ to make the urea concentration less than 2M, followed by two rounds of trypsin digestion as trypsin : protein = 1 : 50 for overnight and trypsin : protein = 1 : 100 for 4 hours respectively.

### Enrichment and deglycosylation

The HILIC enrichment of *N-*glycopeptides was processed as described with minor modifications [37,38]. Briefly, the peptide fractions were equilibrated in 40 μL loading buffer (80% acetonitrile/1% trifluoroacetic acid), then pipetted into a tip containing HILIC beads (4 μm, 100 Å) (Dalian Institute of Chemical Physics, Dalian, China). After centrifugation at 4,000 g for 15 minutes, the glycopeptides were retained in the tip. Then, the HILIC tip was washed with 40 μL of loading buffer three times to remove the residual non-glycopeptides by centrifugation. The enriched glycopeptides were then eluted with 80 μL H_2_O and lyophilized by vacuum centrifugation. Finally, two hundred units of PNGase F (New England Biolabs, Ipswich, England) and PNGase A (Roche, Basel, Switzerland) in 50 μL 40 mM NH_4_HCO_3_ dissolved in H_2_^18^O was applied to the samples and incubated overnight at 37°C for deglycosylation.

### LC-MS/MS

To perform the liquid chromatography-tandem mass spectrometry (LC-MS/MS) analysis, the peptides dissolved in 0.1% formic acid were loaded onto a reversed-phase pre-column (Acclaim PepMap 100, Thermo, Waltham, U.S.A.), then separated using a reversed-phase analytical column (Acclaim PepMap RSLC, Thermo, Waltham, U.S.A.) in solvent B (0.1% formic acid in 98% acetonitrile) at a flow rate of 300 nl/min on an EASY-nLC 1000 UPLC system (Thermo, Waltham, U.S.A.). The gradient was set from 6% to 22% for 48 min, 22% to 35% for 12 min and 85% for 5 min. Then a full-scan mass spectrum was analyzed by Q Exactive^TM^ Plus hybrid quadrupole-Orbitrap mass spectrometer (Thermo, Waltham, U.S.A.) over a mass range of 350–1800 m/z with a resolution of 70,000.

The resulting MS/MS data was processed using MaxQuant software with integrated Andromeda search engine (v.1.4.1.2) against Uniprot *Oryza sativa* database (http://www.uniprot.org/). Deamidation ^18^O on Asn, acetylation on protein *N-*terminal, oxidation on Met and deamidation on Asn and Gln were specified as variable modifications and carbamidomethylation on Cys was specified as fixed modification. 5 modifications per peptide and 5 charges, up to 2 missing cleavages on trypsin/P were allowed. Besides, mass tolerance was set to 10 ppm for precursor ions and 0.02 Da for fragment ions. The thresholds of false discovery rate (FDR) in MaxQuant for peptide, protein and modification site were set at 1% and minimum peptide length was set at 7. The rest of parameters in MaxQuant were set to default values. The probability required for a glycosylation site considered as localized was higher than 75%. The occurrence of *N*-glycosylation modification was defined as being detected in at least two of the three biological replicates.

### Bioinformatic analysis

Gene Ontology (GO) annotation proteome was derived from the UniProt-GOA database (www. http://www.ebi.ac.uk/GOA/). To do the GO analysis, identified protein IDs was converted into UniProt IDs and then mapping to GO IDs. Those identified proteins without an annotation in UniProt-GOA database were processed with InterProScan software for GO functional annotation based on protein sequence alignment method. Then proteins were classified into three categories: biological process, cellular component and molecular function.

Identified proteins domain functional description was annotated by searching against the InterPro domain database of InterProScan (http://www.ebi.ac.uk/interpro/)

Kyoto Encyclopedia of Genes and Genomes (KEGG) database was used to annotate protein pathway. Firstly, using KEGG online service tools KAAS to annotate protein’s KEGG database description. Then mapping the annotation result on the KEGG pathway database using KEGG online service tools KEGG mapper.

Subcellular localization prediction was performed in PSI (http://bis.zju.edu.cn/psi/) plant database with default settings.

Soft motif-x (http://motif-x.med.harvard.edu/) was used to analysis the model of sequences constituted with amino acids in specific positions of modify-21-mers (10 amino acids upstream and downstream of the site) in all protein sequences. And all the database protein sequences were used as background, occurrences set as 50 and other parameters with default.

## Results

### Profiling the *N-*glycosites of rice germinating embryos

**Fig 1:**
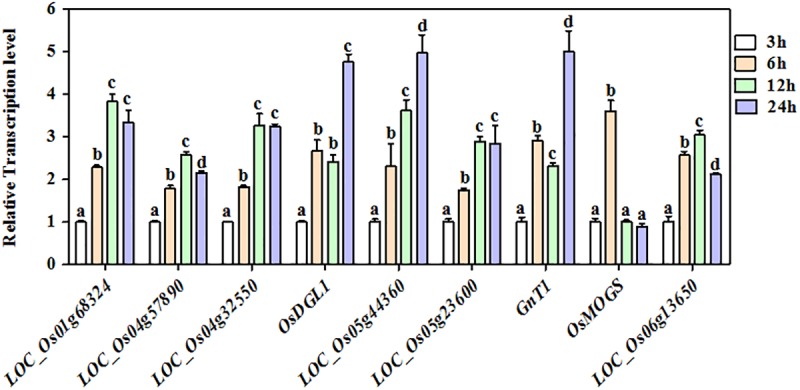
qRT-PCR to check the mRNA level of glycosylation-related genes in the process of rice embryo germination. All values are based on three technical repeats and presented as means±SE. Different characters indicate a statistically significant difference at P<0.05 by t-test.

**Fig 2:**
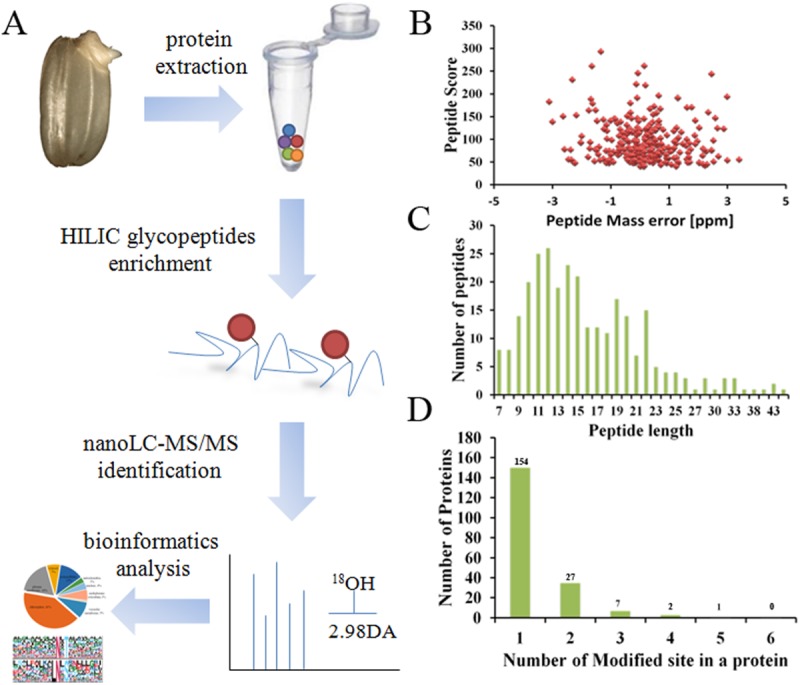
(A) Workflow of the *N-*glycosite mapping of rice germinating embryos; (B) Mass error of all the identified peptides; (C) length distribution of all the identified peptides; (D) glycosylation modification frequency on identified proteins.

**Fig 3:**
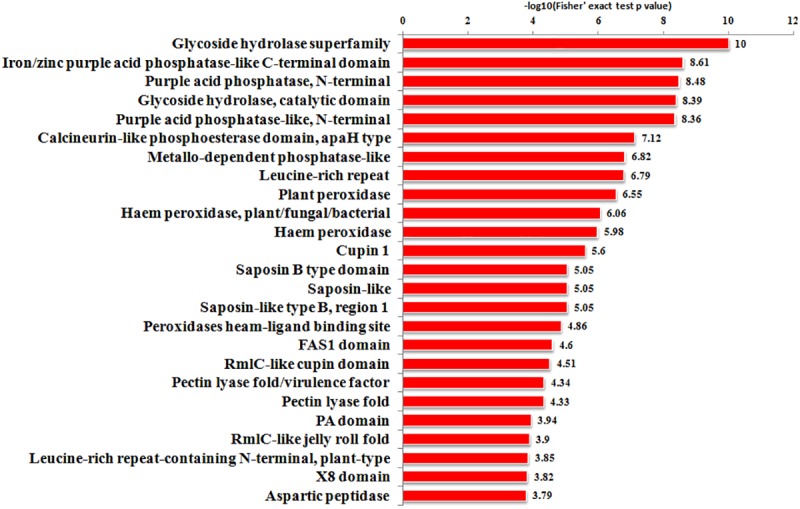
Protein domain enrichment analysis of the identified glycoproteins.

Protein *N*-glycosylation is believed to play key roles in the metabolism of various biological processes, including seed germination. It has been reported that the metabolism in rice germinating embryos is robustly reactivated at around 24 HPI (Hours Post Imbibitions) with the highest ATP content [34,39,40]. Hence, 24 HPI has been used as a representative time point for the proteome analysis with metabolism-related PTMs in rice germinating embryos. Prior to the proteome analysis, we examined the transcriptional level of 9 known or putative protein glycosylation complex genes in a time-course of embryo germination. The expression levels of all the tested genes were elevated with the time of water imbibitions. Particularly, 6 of the genes, including *OsDGL1* and *GnT1*, reached the plateau at 24 HPI, indicating that 24 HPI might be a key stage of protein *N*-glycosylation (Fig 1). To dissect the potential roles of protein *N*-glycosylation in rice germination, an *N-*glycosites mapping analysis of Nipponbare (*Oryza sativa* L. *ssp japonica*) embryos at 24 HPI was conducted by employing a MS-based approach with three biological replicates. Our experimental procedures consists four typical steps as shown in Fig 2A: protein extraction, HILIC glycopeptides enrichment, nanoLC-MS/MS identification and bioinformatics analysis. To ensure a high quality of the MS data, we analyzed the mass errors of each detected peptide and set a cut-off of 4 ppm for those to be further processed (Fig 2B). As a result, a total of 242 glycosites within 191 glycoproteins were identified in the current study with a FDR (False Discovery Rate) less than 1% (Table 1). The length of the identified glycopeptides was ranged from 7 to 44 residues with an average number of 16 (Fig 2C). Meanwhile, 80.6% of the glycoproteins carried one glycosysite, 7.7% carried two sites, while the remaining 11.7% had three or more glycosysites (Fig 2D). A functional domain enrichment analysis of the glycoproteins revealed that proteins harboring “glycoside hydrolase superfamily”, “Iron/zinc purple acid phosphatase-like C terminal”, “Purple acid phosphatase, N-terminal” and “glycoside hydrolase catalytic” domains were the top 4 significantly enriched types when the rice genome was used as a reference (P<0.001), indicating that glycosylation may preferentially occur on proteins with one of these domains (Fig 3). In rice, a long list of transcription factors has been reported to regulate seed dormancy and germination (http://www.ricedata.cn/gene/). To our surprise, none of the glycoproteins identified here corresponded to transcription factors in the plant transcription factor database V3.0 (http://planttfdb.cbi.pku.edu.cn/)[41]. In comparison with the high percentages of glycosylated enzymes, the result suggested that protein glycosylation regulates rice embryo germination primarily by modifying protein activities, while imposes less or not effects in the mRNA transcription of downstream genes.

**Table 1:** Some selected examples of the identified glycoproteins in rice germinating embryos.

Protein accession	Gene ID	Protein annotation	Position	Modified sequence
B9EVS6	LOC_Os01g21034	Pectinesterase	82	_SLPDVICGTVN(de)R_
Q5QMT0	LOC_Os01g32364	Beta-glucosidase 1	96	_TPGEIANN(de)ATADVTVDEYHR_
Q8GT94	LOC_Os01g59440	Floral organ regulator 1	77	_LDLGNLN(de)LSGHLVPELGK_
Q67IX6	LOC_Os02g01010	Protein disulfide isomerase-like 1–4	315	_QILLFVVAN(de)ESSK_
Q75IZ5	LOC_Os03g30000	glycosyltransferase 8 domain containing protein	529	_YVN(de)FTHPYVR_
Q10E34	LOC_Os03g56270	receptor protein kinase CLAVATA1 precursor	559	_ILNYLN(de)LSR_
Q0JDC5	LOC_Os04g33740	Beta-fructofuranosidase, insoluble isoenzyme 2	189	_PGHNPVIVPEGGIN(de)ATQFR_
Q7X6F6	LOC_Os04g54810	beta-D-xylosidase	521	_TSLLLPGQQPQLVSAVAN(de)ASR_
Q7XQ88	LOC_Os04g57890	Dolichyl-diphosphooligosaccharide—protein glycosyltransferase subunit STT3B	571	_TVIVDNNTWN(de)NTHIATVGR_
Q0DJC5	LOC_Os05g23600	Dolichyl-diphosphooligosaccharide—protein glycosyltransferase subunit 1A	300	_DEIGN(de)ISTSHLWSDSK_
Q6I5I5	LOC_Os05g45430	TOO MANY MOUTHS precursor	363	_MYHLN(de)LSK_
Q9LX04	LOC_Os06g01490	monocopper oxidase	295	_FVN(de)ESLWTK_
Q5WA72	LOC_Os06g06790	Protein disulfide isomerase-like 1–5	151	_GFPTVLLFVN(de)GTEHQFTGLHTK_
Q653V7	LOC_Os06g46284	glycosyl hydrolase, family 31	378	_FVVIIDPGINVN(de)TTYGTFVR_
Q0D9Q0	LOC_Os06g49100	retrotransposon protein, putative, unclassified, expressed	180	_N(de)FTYEDNFFSSR_
Q0D9G9	LOC_Os06g50300	heat shock protein	110	_ELISN(de)ASDALDK_
Q8H3S1	LOC_Os08g23180	fasciclin-like arabinogalactan protein 8 precursor	218	_NFAGLLASNADVYSNIN(de)ATK_
Q6Z3T9	LOC_Os08g39550	polygalacturonase inhibitor 2 precursor	293	_GLGILN(de)LSR_
Q0IQP9	LOC_Os12g01700	inactive receptor kinase At2g26730 precursor	290	_LN(de)GTIPDR_
Q2QV45	LOC_Os12g14070	DnaK family protein	658	_DAMAALNEEVMQIGQAM(ox)YN(de)QQPN(de)

**Fig 4:**
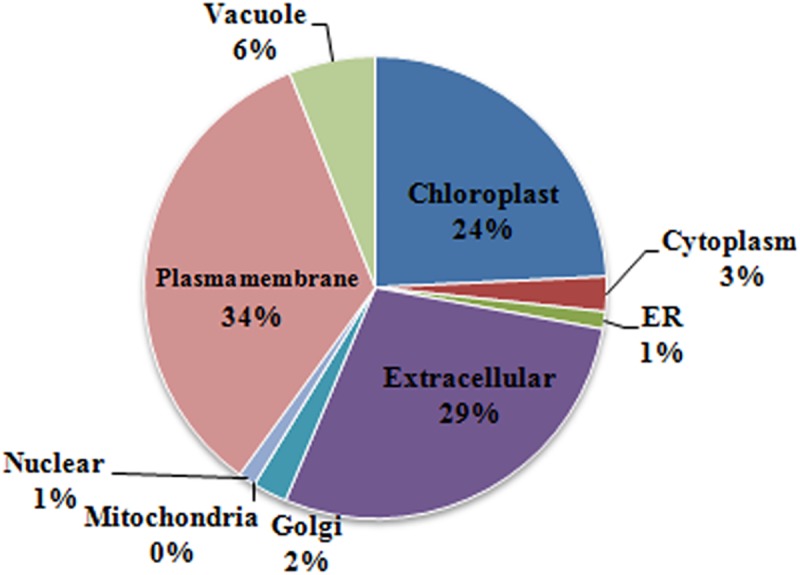
Distribution of glycoproteins in different subcellular compartments.

To gain an insight into the glycoprotein distribution across subcellular compartments, the amino acid sequence of glycoproteins were used to search against the plant database of PSI (Plant Subcellular location Integrative predictor) (http://bis.zju.edu.cn/psi/) [42]. By using a joint-approach of group decision making strategy and machine learning methods, PSI integrates 11 specialized predictors, including CELLO and Wolf PSORT, to give an optimized result of subcellular localization prediction. As shown in Fig 4, extracellular was the largest group of glycoprotein destination, which accounted for 29% of the proteins analyzed. This observation is in accordance with the previous reports that glycoproteins are extensively involved in the protein secretion [29]. We also found that 34% and 24% of the glycoproteins potentially located in plasma membrane and chloroplast respectively, whereas the remaining 7 subcellular compartments like nuclear, cytoplasmic and mitochondria taken together occupied a percentage of 13. It is interesting that 27% of glycoproteins are directed to the plasma membrane, but only 2% to the chloroplast in the *Brachypodium distachyon* seedling, while the results from maize endosperm were quite controversial with a ratio of 33% and 3% in chloroplast and plasma membrane respectively [17,28]. The discrepancy of subcellular distribution might be ascribed to the differences in cell types, protein extraction methods and prediction tools used. Moreover, SignalP 4.1 (http://www.cbs.dtu.dk/services/SignalP/) was applied to predict the presence of signal peptides on our glycoproteins. As a result, 147 out of the 191 glycoproteins were found to harbor a signal peptide, indicating their potential roles in protein secretion (S2 Table).

### Features of the sequence flanking the glycosites

**Fig 5:**
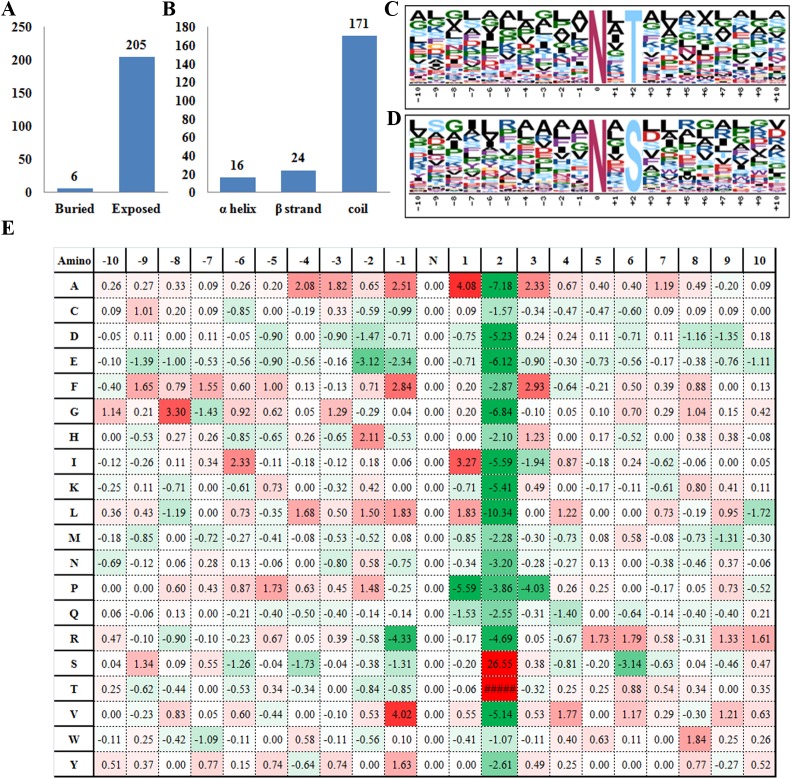
(A) Number of glycosites located on the buried or exposed region of the proteins; (B) Number of glycosites in different secondary structures; (C-E) Motif-X analysis of the over-represented motifs around the glycosites, (C) [NxT], (D) [NxS] (x can be any amino acids except proline). (E) Motif analysis of all the identified glycosites.

To investigate the featured secondary structures and conserved motifs flanking the glycosites, we extracted a 13 amino acid-long, glycosite-centered sequence for each site. Due to the location in the 5’ or 3’ end of the protein sequences, 31 glycosites failed to provide a valid extracted sequence and consequently 211 extracted sequences were applied for further analysis. By using the online tool NetsurfP (http://www.cbs.dtu.dk/services/NetSurfP/) [43], 205 glycosites were predicted to be on the exposed surface of the protein, the remaining 6 lay buried within the protein structures (Fig 5A). A surface location is expected for these sites, in order to allow access for the glycosylation catalyzing enzyme(s). 16 (7.6%), 24 (11.4%) and 171 (81.0%) of the glycosites were found to be located in α helix, β strand and coil protein secondary structures, respectively (Fig 5B). The ratios we observed in rice is similar to the previous report on *Brachypodium distachyon* leaves, suggesting that glycosylation favors more amino acid sites in coils other than α helix and β strand. Furthermore, Motif-x analysis was conducted for the glycosite flanking sequences (http://motif-x.med.harvard.edu/)[44] and resulted in the identification of two over-represented motifs [NxT] and [NxS] (x can be any amino acids except proline), which accounted for 65.1% and 34.9% of the set of motifs respectively (Fig 5C and 5D). Meta analysis of public glycoproteome data from 7 model organisms has deduced that 97–99% of the *N*-glycosylation should occur on [NxS/T] motif, in which the frequency for T is around two times higher than S [29]. Our observation in rice is consistent with the previous reports, suggesting a highly conserved sequence context for protein glycosylation among different species. In addition to the specified sites mentioned above, the frequencies of each residue present in the flanking sequence is depicted in Fig 5E. Taking the glycosite as position zero, P is unlikely to appear in +2 and +3 either. On the contrary, A has a high possibility to show up in -1, +1 and +3 position. The -1 position also seems to favor more L, V, A and F than other amino acids. The featured amino acid context may provide valuable clues for the prediction of glycosites on unknown proteins.

### Functional enrichment analysis of the glycoproteins

**Fig 6:**
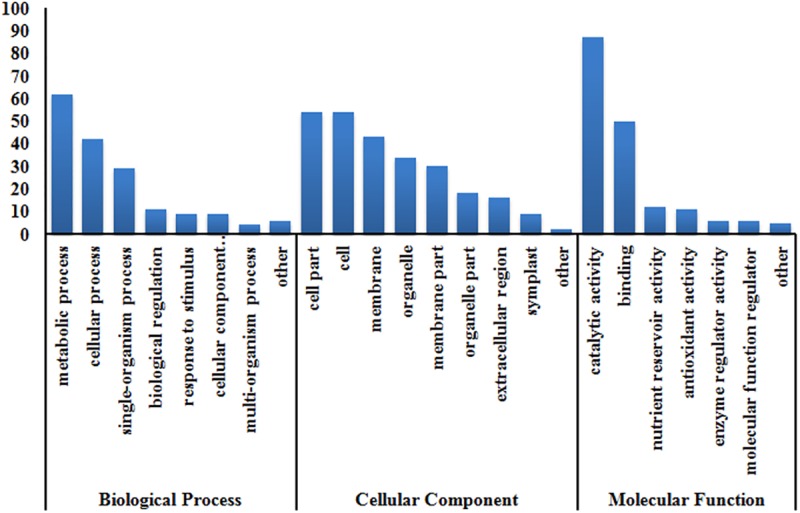
GO classification of the identified glycoproteins in terms of biological process, cellular component and molecular function.

To explore the potential functions of the identified glycoproteins, a GO (Gene Ontology) analysis was conducted in the vocabulary of “biological process”, “cellular component” and “molecular function”. In terms of “biological process”, proteins related to metabolism such as carbohydrate metabolic and primary metabolic processes were most significantly enriched for glycosylation, which is in agreement with the scenario that vigorous metabolism is required to supply energy for embryo germination. In support to the enriched metabolic pathway process, we observed that the glycoproteins were enriched in enzymes with hydrolase activity, nutrient reservoir and catalytic activities under the “molecular function” perspective. Meanwhile, in terms of “cellular component”, glycosylation is preferentially occurred on proteins located in vacuole and ER, though the absolute number for the two categories is not high (Fig 6).

When a similar analysis was based on KEGG (Encyclopedia of Genes and Genomes) [45], 6 different pathways, including proteins processing in ER (osa04141), starch and sucrose metabolism (osa00500), *N-*glycan biosynthesis (osa00510) and metabolic pathways (osa01100), were highlighted (S1–S6 Figs). It seems that proteins involved in metabolism process during seed germination are subjected to the protein glycosylation regulation because related pathways were found to be enriched by both our GO and KEGG analysis. Moreover, ER is proposed to be the nursery for protein glycosylation processing, while *N-*glycan biosynthesis pathway supplies glycan moieties for protein glycosylation. The enrichment of these two pathways implied that enzymes catalyzing glycosylations may also be subjected to the regulation of protein glycosylation.

### Protein-protein interaction (PPI) network of glycoproteins

**Fig 7:**
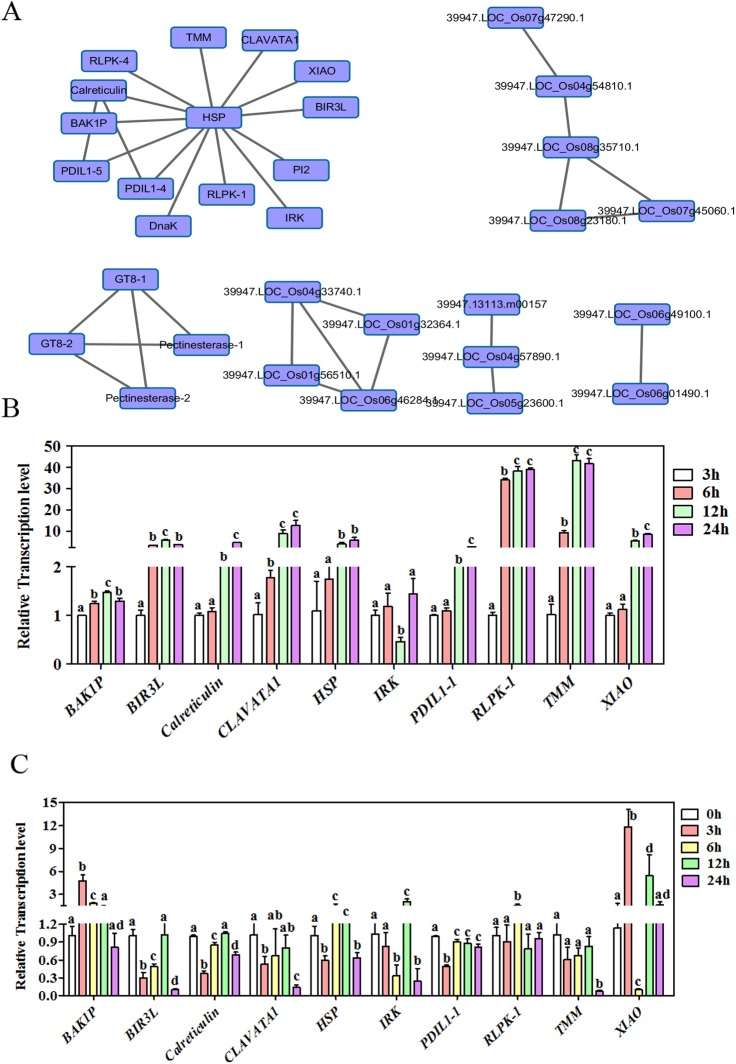
(A) PPI analysis of the glycoproteins. (B-C) qRT-PCR to check the mRNA level of some glycoproteins genes in the process of rice embryo germination (B) and in response to BRs treatment (C). All values are based on three technical repeats and presented as means±SE. Different characters indicate a statistically significant difference at P<0.05 by t-test.

PPI prediction helps to determine the components belonging to the same functional protein complex or enzyme-substrate relationship. In this study, the STRING (Search Tool for the Retrieval of Interacting Genes/Proteins) version 10.0 (http://string-db.org/) was employed to analyze the potential interaction relationships of the glycoproteins [46]. We set the confidence score to 0.7 to assure a high reliability, and visualized the results by Cytoscape software [47]. The results displayed a complicated network with 32 nodes (proteins) and 33 edges (interaction relationships) (Fig 7A). Network group A consisting 14 nodes and 15 edges attracted our particular attention. The network was centered by an unknown heat shock protein (HSP) (LOC_Os06g50300), while the HSP interacting proteins included 8 kinases, 2 PDILs enzymes (Protein Disulphide Isomerase-Like), along with calreticulin, polygalacturonase. To explore the relevance of these genes with seed germination, we examined their mRNA expression dynamics in Nipponbare germinating embryos at 3 HPI, 6 HPI, 12 HPI and 24 HPI. Due to the technique difficulties in RNA isolation from the dry and tiny embryos, we did not include the 0 HPI samples for this analysis, thus 3 HPI was taken as a control to present the early germination status. Of the 13 genes investigated, ten were up-regulated by at least two fold between 3 and 24 HPI (Fig 7B). In particular, the expression of *LOC_Os02g11930* and *LOC_Os05g45430* were over 30 time elevated, suggesting their potential functions in the germination process. Several members of this protein network are thought to be functionally related to BRs signaling. For example, LOC_Os01g59440 is annotated to be a close homolog of BAK1 (BRI1-Associated receptor Kinase), which acts as a kinase of BRs receptor; XIAO (LOC_Os04g48760) was reported to be a BRs signaling component controlling rice organ size. qRT-PCR analysis further revealed that *XIAO*, *LOC_Os05g44770*, *LOC_Os06g36270* and *LOC_Os10g33040* were highly responsive (>5 fold change) to BRs treatment (Fig 7C), which was taken to indicate that glycosylation on BRs signaling components might be mechanistically important in seed germination regulation. The other potential PPI networks may be related to cell wall degradation, saccharine metabolism.

## Discussion

### *N-*glycosites profiling in plants

*N-*glycosylation is one of the most prominent and abundant protein posttranslational modifications. It is estimated that over 50% of the proteins are modified by covalent attachment of sugar molecules at some point in their life cycle [48]. Unlike in mammals, *N-*glycoproteomic profiling in plants is still in its infancy. To date, around 1000 *N-*glycosylated proteins have been identified with high confidence in the model plant *Arabidopsis thaliana* [26,27,29]. In grapevine (*Vitis vinfera*), Melo-braga et al (2012) carried out a quantitative analysis of the glycoprotoeme's response to pathogen infection, realizing over 300 glycosites and identifying at least one differentially glycosylated disease resistance response product [24]. In 2014, a multi-lectin approach was applied to enrich the *N-*glycoproteins of tomato (*Solanum lycopersicum*) fruit for MS identification. The multi-lectin approach revealed 318 putative *N*-glycosylation sites on 230 *N-*glycoproteins as well as 17 *N-*glycan structures, which substantially increased the glycoproteomic coverage when compared with the single lectin method [25]. In cereals, barley (*Hordeum vulgare*) aleurone layers maintained *in vitro* respond to gibberellic acid by secreting an array of proteins, which provide a unique system for the analysis of plant protein secretion. *N-*glycosylation analysis in gibberellic acid-induced aleurone layers identified 73 glycosylation sites in 65 aleurone layer proteins, with majority were found in extracellular and intracellular fractions [20]. *N-*glycosylation is also involved in food allergy, as several identified, *N-*glycosylated albumin proteins in wheat shared high sequence similarities with known food allergens [22]. Glycoproteomic characterization has also been attempted in cotton and various bio-fuel grasses and trees [17,19,21,23,28]. To the best of our knowledge, the identification of 242 glycosites on 191 proteins in the current study represents the largest scale profiling of glycoproteome in crops so far (S3 Table).

### Glycosylation on starch and sucrose metabolism proteins

Starch and sucrose metabolism are central metabolic events for rice seed germination. During the early germination of seeds, the establishment of plants from embryos depends entirely upon the carbohydrate reserves remobilized from starch granules stored in endosperm. Glucose derived from the endosperm starch degradation is transferred to the scutellum where it is converted into sucrose. To maintain the homeostasis of sucrose in the germinating embryo, the imported sucrose is transiently synthesized into starch before the protrusion is completed [49,50]. Here, the KEGG pathway analysis of the glycoproteins revealed that many of the glycosylation events affected proteins involved in starch and sucrose metabolism, suggesting that protein glycosylation is involved in the embryo germination by regulating carbohydrate metabolism. At least 7 proteins in this pathway were found to be glycosylated, including α- and β-glucosidase, hexosyltransfearase, β-fructofuranosidase and pectinesterase, among which CIN2/GIF1 (LOC_Os04g33740), encoding a β-fructofuranosidase (also named as cell-wall invertase), attracted our particular attention. It has been known that β-fructofuranosidases catalyze the breakdown of sucrose-6P into α-D-Glucose-6P and β-D-Fructose, which is a key step of the sucrose metabolism. Owing to the accumulated mutation in the gene’s regulatory sequence through domestication, *CIN2/GIF1* of cultivars showed a restricted expression pattern in the ovular vascular trace during grain-filling compared to the wild rice allele. *gif1* mutant as well as the constitutively ectopic expression lines of *GIF1* displayed smaller grains and chalkiness, whereas the restricted over-expression of *GIF1* in ovular vascular trace increased grain production. It was pointed out that *GIF1* is broadly expressed in the grain of wild rice, which might promote the energy metabolism for plant. In contrast, the restrictive expression pattern of *GIF1* cultivars alleles in the ovular vascular bundle facilitate sucrose unloading favoring grain-filling, on the other hand avoid the cost of energy metabolism in other unrelated tissues, which ultimately improves rice yield [51]. It seems that glycosylation on β-fructofuranosidase is conserved among species, as another β-fructofuranosidase INV1 was also found to be glycosylated in *Brachypodium distachyon* [28]. For the *A*. *thaliana* cell wall invertase 1-FEH IIa, *N*-glycosylation has been shown to be critical for maintaining both enzyme stability and its optimal conformation [52]. We therefore hypothesized that glycosylation modification is involved in the sucrose metabolism possibly by regulating the enzyme activity of key regulators such as CIN2/GIF1. Glycosylation also affected glucosidase involved in sucrose degradation, hexosyltransferase related to starch substrate transportation and pectinesterase involved in amino and nucleotide sugar metabolism, implying that glycosylation is extensively participated in the starch and sucrose metabolism during embryo germination.

### Glycosylation on BRs signaling proteins

As the sixth class of plant hormones, BRs have been known to play critical roles in various biological processes including seed germination. During seed germination, water imbibition activates the phosphorylation of several BRs signaling components, and BRs treatment could significantly promote rice seed germination, but not postgerminative growth [39]. It was reported that the interaction of BRs signaling protein BIN2 (Brassinosteroid INsensitive2) and ABA signaling protein ABI5 (ABscisic acid Insensitive 5) antagonistically coordinates seed germination in Arabidopsis [53]. In the current study, a PPI analysis of germinating embryo glycoproteins revealed a complicated network which covered several BRs signaling related proteins. *XIAO* encoding a leucine rich repeat kinase has been known to regulate the signaling and homeostasis of BRs. *xiao* mutant displayed typical BRs defection-related phenotypes such as dwarfism, erect leaves and smaller organ size due to the reduced cell division rate. XIAO may be a key regulator bridging BRs and cell-cycle regulation in controlling organ growth [54]. Moreover, our qRT-PCR results also showed that *XIAO* and three other receptor-like kinase genes was highly responsive (>5 folds) to BRs treatment, indicating its potential roles in BRs signaling. On the basis of sequence homology, *LOC_Os01g59440* is a homolog of *OsBAK1*, while *LOC_Os09g04490* is a closely related to Arabidopsis *BIR3*. As a co-receptor of BRs, BAK1 binds with BRs receptor BRI1 in the presence of BRs to form a BRI1-BR-BAK1 complex, which is the core component in BRs signaling. OsBAK1 has been documented as being involved in rice architecture and innate immunity [55,56]. BIR3 was identified as a receptor-like kinase interactive with BAK1, but its biological function remains unknown [57]. The abundance of *LOC_Os01g59440* and *LOC_Os09g04490* transcript was also responsive to BRs treatment. The implication of the present results is that the glycosylation of BRs signaling components or BR-responsive proteins may well represent an important regulatory mechanism during germination. Surprisingly, the transcription of the gene encoding the heat shock protein LOC_Os06g50300, which lies at the center of the PPI network, was not influenced by the BRs treatment, even though it did potentially interact with several BRs signaling components. The assumption was therefore that LOC_Os06g50300 acts as a molecular chaperone to stabilize BRs signaling components during germination.

### Glycoproteins related to cell wall modification and remodeling

Cell wall is a major component deciding the mechanical strength and morphology of plant cells. It has been known that robust cell wall synthesis and remodeling is accompanied with the radical protrusion and new organ formation during rice embryo germination [58]. In the current study, 4 pectinesterases were indentified to be glycosylated. pectinesterases are ubiquitous enzymes for cell wall modification. It catalyzes the de-esterification of pectin, which is a structural heteropolysaccharide in the primary cell wall of plants, into pectate and methanol. De-esterification of pectin causes proton release and activates the cell wall hydrolases, which eventually could loosen the cell wall for plant growth. We also identified 3 polygalacturonases, whose function is involved in the degradation of pectin backbone polymer polygalcturonan. In Arabidopsis, *PGX1* (*POLYGALACTURONASE INVOLVED IN EXPANSION1*) encoding a putative polygalacturonase were reported to regulate apoplastic pectin degradation. PGX1 displayed *in vitro* polygalacturonase activity. Overexpressing or lacking *PGX1* altered the total polygalacturonase activity, pectin molecular mass, and wall composition, which finally led to compromised hypocotyls elongation and floral organ patterning [59]. In contrast to the function of polygalacturonase, glycosyltransferases are thought to regulate the biosynthesis of pectins in plants. In Arabidopsis, the mutants of a glycosyltransferase family 8 gene exhibited 25% reduction of the pectin content [60]. It is interesting that the current study revealed 3 glycosylation sites on 2 of the glycosyltransferase family 8 members (LOC_Os03g30000 and LOC_Os03g56620). Moreover, other cell-wall modification-related glycoproteins include β-glucosidase (LOC_Os01g56510), β-D-xylosidase (LOC_Os04g54810), xylose isomerase (LOC_Os07g47290), and so on. Given the reported extensive involvement of glycosylated enzymes in the cell wall-related events [23,25,28], our results suggested that *N*-glycosylation may play important roles in cell wall modification and remodeling.

### Glycosyproteins regulating protein processing in ER

In the eukaryotic cells, the ER is an organelle in the forms of flattened, membrane-enclosed sacs. ER is the occasion for protein processing, including protein synthesis, folding, translocation of nascent polypeptides for secretion or insertion into the membrane as transmembrane proteins [61]. Our KEGG pathway analysis revealed that glycoproteins were enriched for the pathway of “protein processing in ER” (S6 Fig). Most of the secretory proteins and membrane proteins enter the ER lumen and become glycosylated in there for the protein maturation. OST (oligosaccharyltransferase) is the enzyme responsible for catalyzing the transfer of Glc3Man9GlcNAc2 to the growing polypeptide chain, while the function of glucosidase I (GlcI) is to trim the terminal glucose residue. Interestingly, we found that 2 OSTs (OST1A and OST1B) as well as a GlcI were glycosylated. Proper protein folding is another critical step for the protein maturation. Protein folding is a high-fidelity process assured by a complicated QC (quality control) system, which could sense the misfolded proteins and execute refolding or degradation on them. Calnexin/calreticulin cycle is the major component of the QC system in ER. Calnexin is a Type I integral membrane protein. Its cytoplasmic tail is responsible for protein folding as well as post-translational modifications. Meanwhile, calreticulin, an ER localized protein with high capacity of Ca^2+^ buffering, is involved in the substrate recognition. By interacting with the sugar residues on the glycoprotein, calnexin/calreticulin complex together with ERp57 facilitate the formation of disulfide bond for protein folding [61]. As we expected, a calreticulin protein (B9FWE4) was found to be glycosylated in our data (S2 Table). In addition to the proteins mentioned above, we also identified 3 glycosylated PDIs (Protein Disulfide Isomerase) which are the members of the QC complex (S2 Table). A previous study in barley indicated that glycosylated PDIs may participate in the correction of the misfolded proteins [20].

## Conclusion

This study identified 242 glycosites on 191 proteins from the rice germinating embryos, representing the largest scale profiling of glycosites in crops. *N-*glycosylation preferentially occurred on proteins related to starch and sucrose metabolism, BRs signaling, cell wall modification and remodeling, protein processing in ER, suggesting that *N-*glycosyaltion is involved in embryo germination by regulating carbohydrate metabolism, and glycosylation-mediated BRs signaling may be a key mechanism regulating rice embryo germination.

### Availability of supporting data

The mass spectrometry proteomics data have been deposited to the ProteomeXchange Consortium [62] *via* the PRIDE partner repository with the dataset identifier PXD004417.

## Supporting information

S1 Fig Enriched KEGG pathway of starch and sucrose metabolism. (TIF)

S2 Fig Enriched KEGG pathway of *N-*glycan biosynthesis. (TIF)

S3 Fig Enriched KEGG pathway of glycan degradation. (TIF)

S4 Fig Enriched KEGG pathway of glycosphingolipid biosynthesis. (TIF)

S5 Fig Enriched KEGG pathway of phenylpropanoid biosynthesis. (TIF)

S6 Fig Enriched KEGG pathway of protein processing in ER. (TIF)

S1 Table Primers used in this study. (XLSX)

S2 Table Glycosylation proteins, peptides and sites of rice germinating embryos at 24 HPI. (XLSX)

S3 Table Reported glycoproteomic cases in plants. (XLS)
