# Supplementary figures and images for: Mapping the N-linked glycosites of rice (Oryza sativa L.) germinating embryos
Source: PLoS One. 2017 Mar 22;12(3):e0173853. doi: 10.1371/journal.pone.0173853 (PMC5362090; doi:10.1371/journal.pone.0173853)

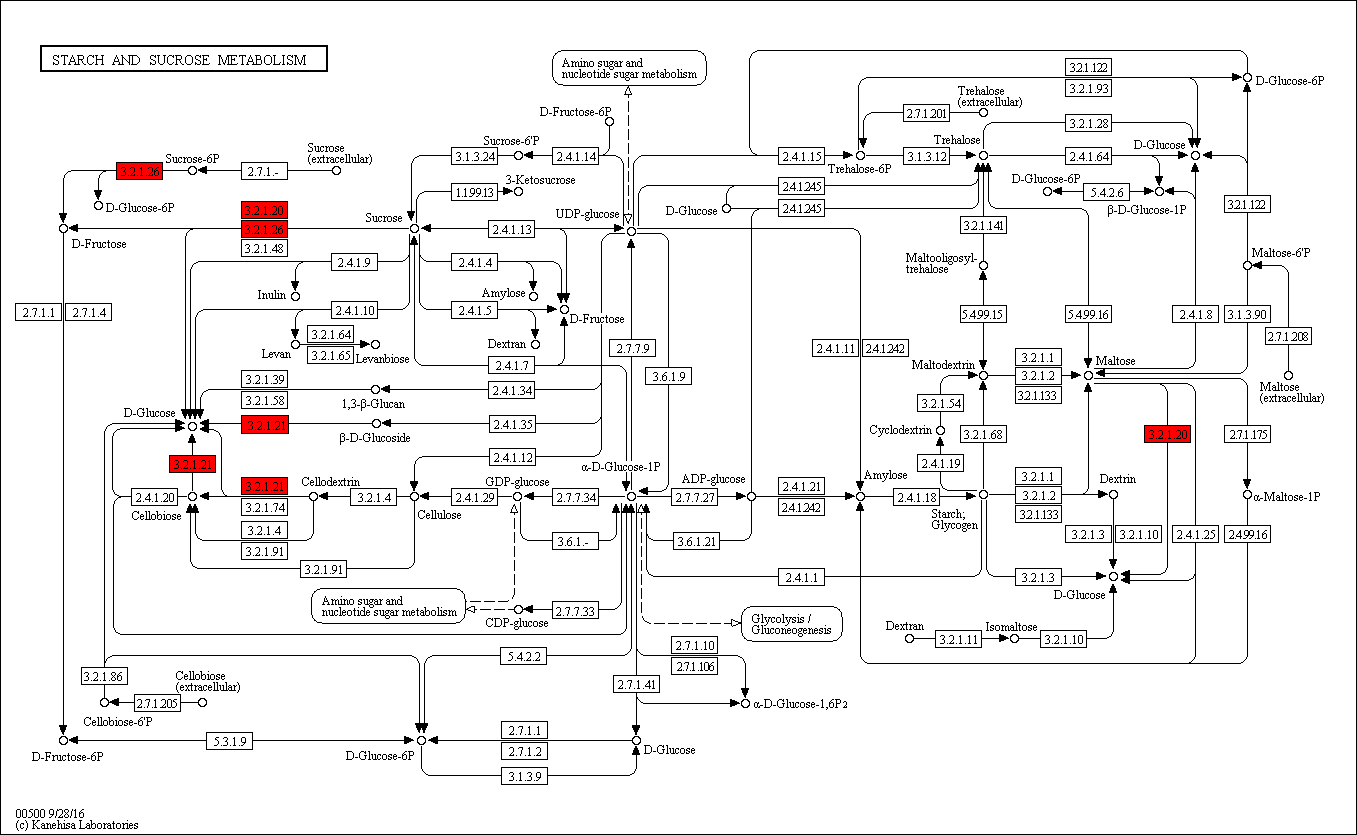

Supplement: S1 Fig — (TIF) [file pone.0173853.s001.tif]

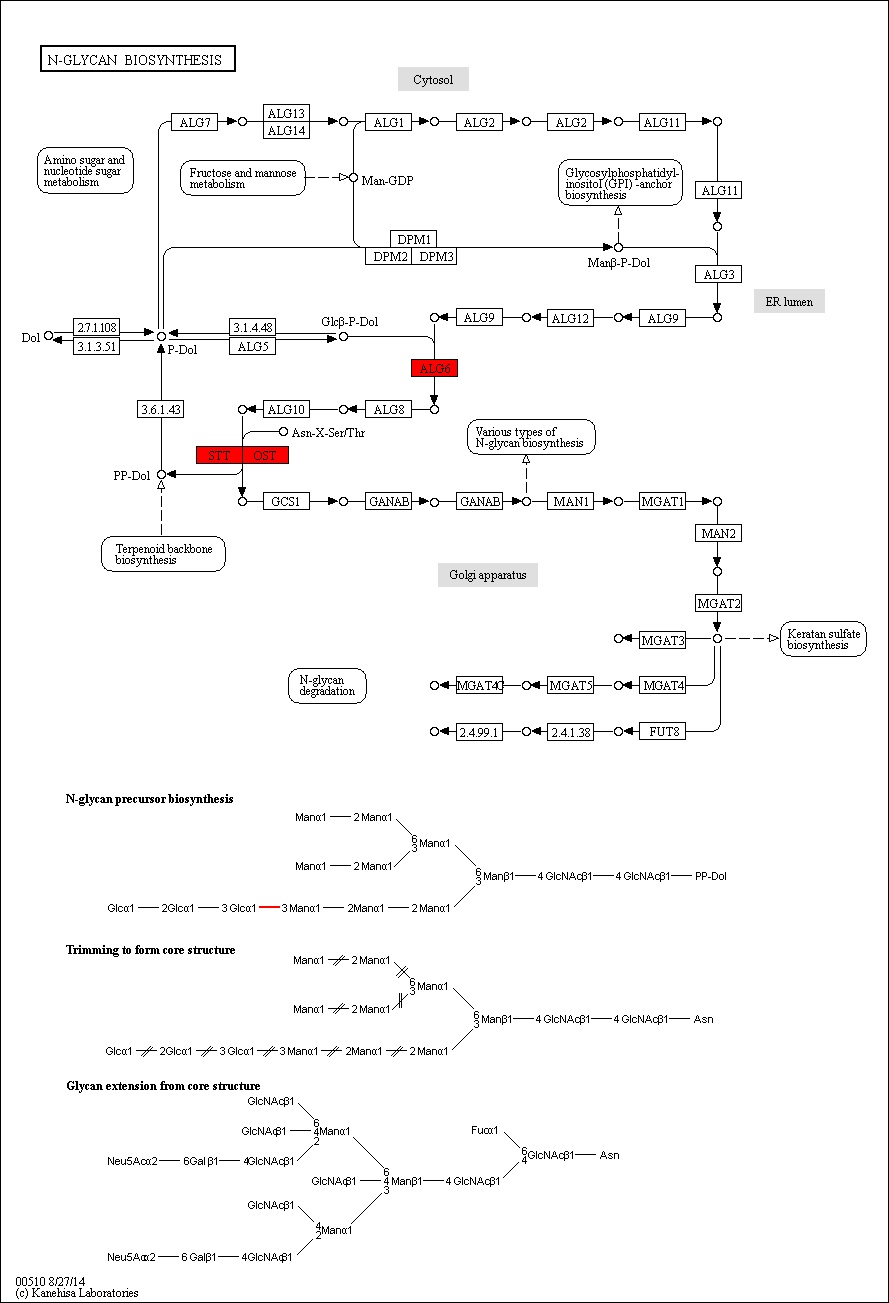

Supplement: S2 Fig — (TIF) [file pone.0173853.s002.tif]

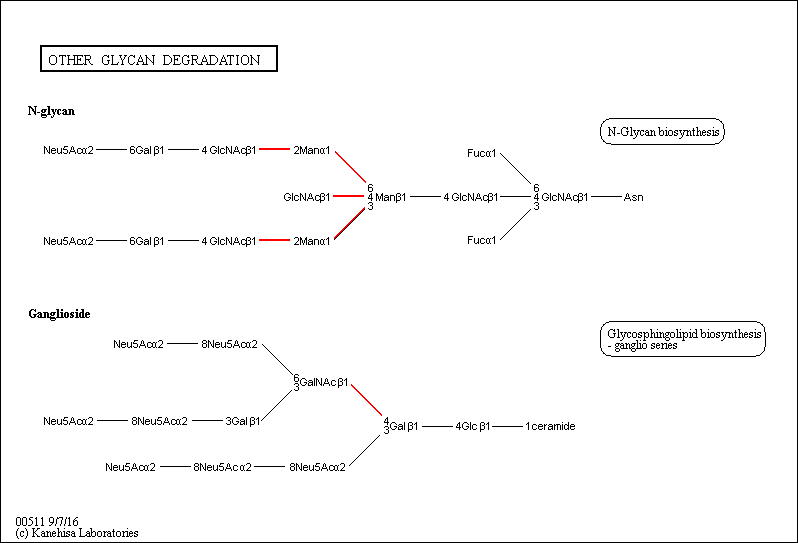

Supplement: S3 Fig — (TIF) [file pone.0173853.s003.tif]

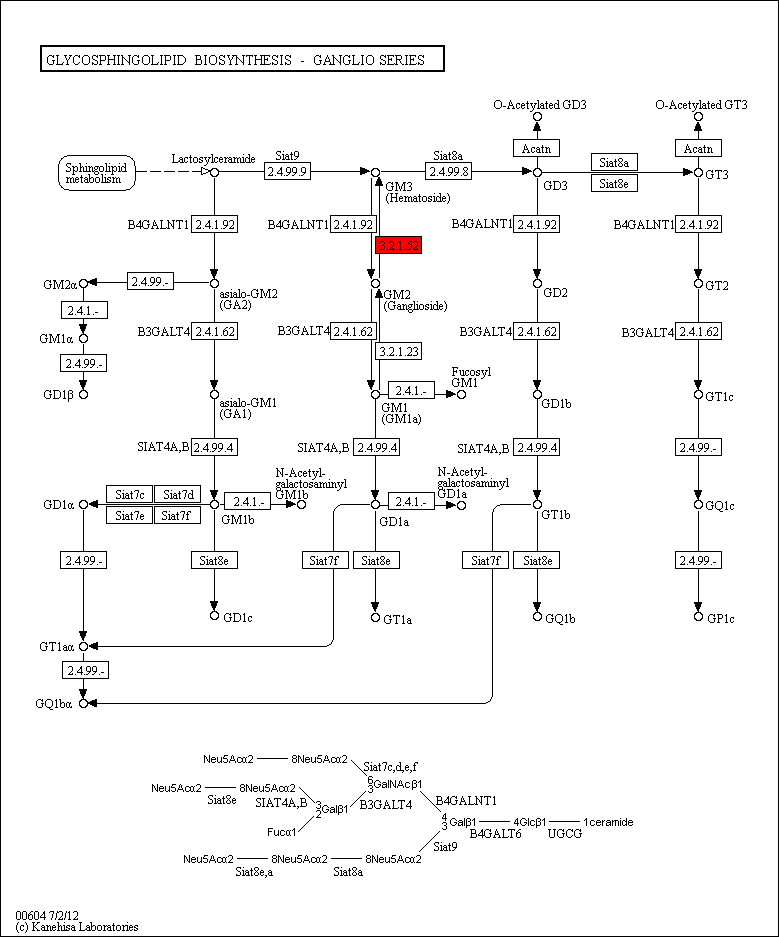

Supplement: S4 Fig — (TIF) [file pone.0173853.s004.tif]

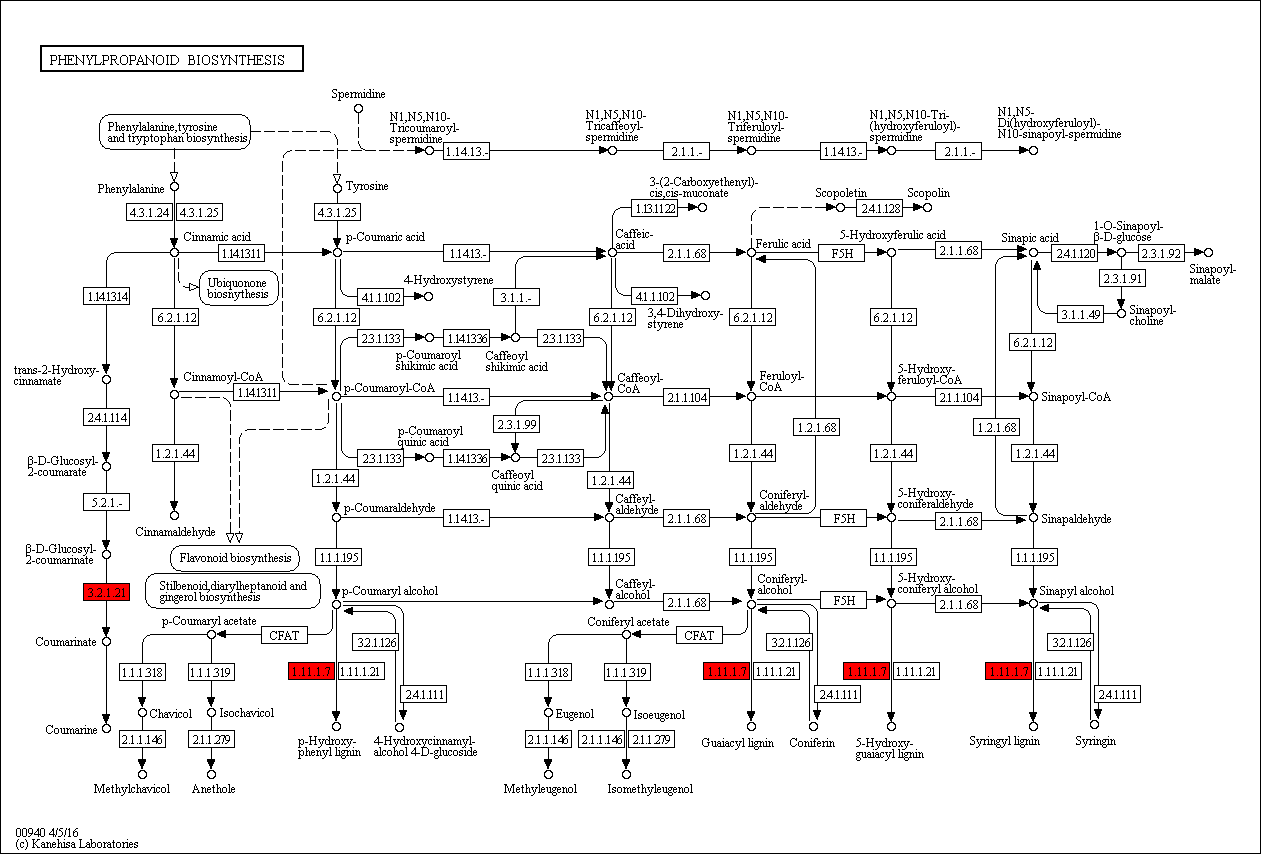

Supplement: S5 Fig — (TIF) [file pone.0173853.s005.tif]

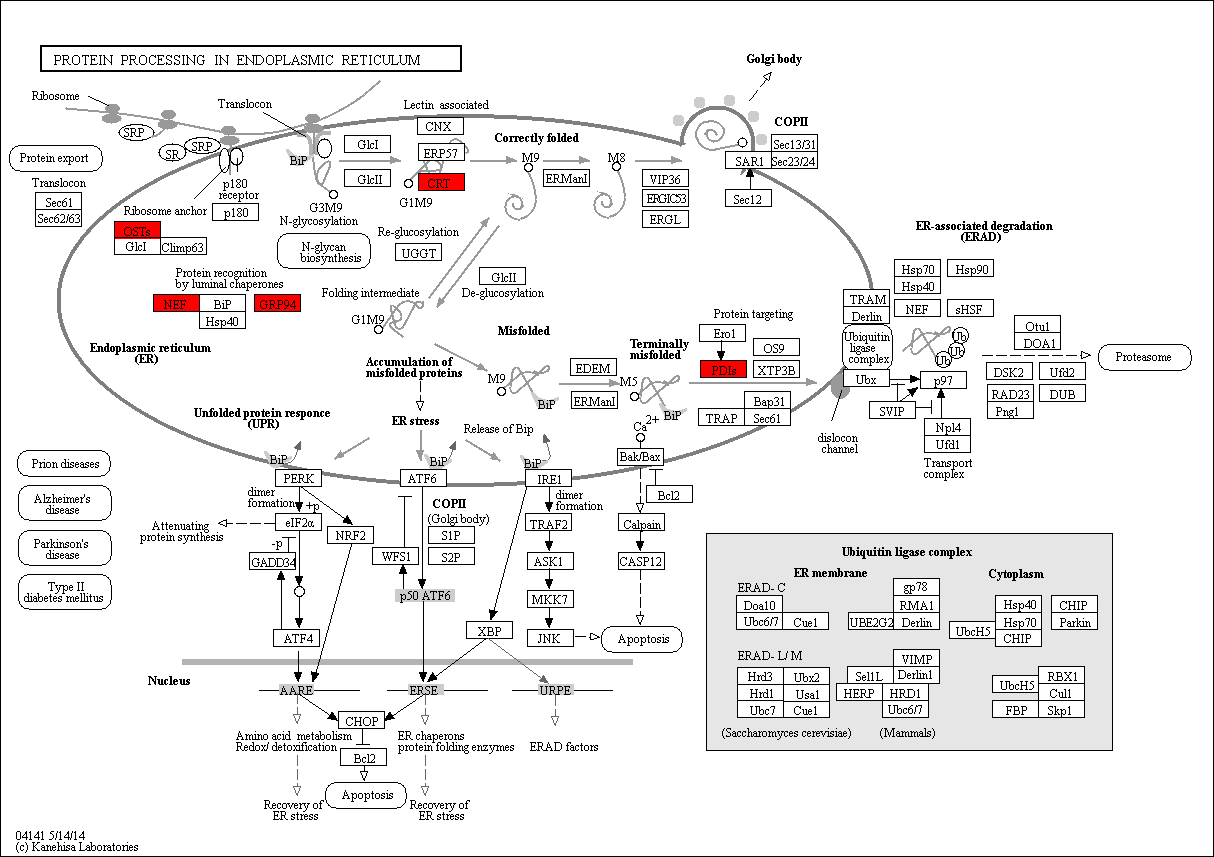

Supplement: S6 Fig — (TIF) [file pone.0173853.s006.tif]
